# Exonic deletions in *IMMP2L* in schizophrenia with enhanced glycation stress subtype

**DOI:** 10.1371/journal.pone.0270506

**Published:** 2022-07-01

**Authors:** Akane Yoshikawa, Itaru Kushima, Mitsuhiro Miyashita, Kazuhiro Suzuki, Kyoka Iino, Kazuya Toriumi, Yasue Horiuchi, Hideya Kawaji, Norio Ozaki, Masanari Itokawa, Makoto Arai

**Affiliations:** 1 Schizophrenia Research Project, Department of Psychiatry and Behavioral Sciences, Tokyo Metropolitan Institute of Medical Science, Setagaya, Tokyo, Japan; 2 Department of Psychiatry, Tokyo Metropolitan Matsuzawa Hospital, Setagaya, Tokyo, Japan; 3 Department of Psychiatry, Nagoya University Graduate School of Medicine, Nagoya, Aichi, Japan; 4 Medical Genomics Center, Nagoya University Hospital, Nagoya, Aichi, Japan; 5 Department of Psychiatry, Takatsuki Clinic, Akishima, Tokyo, Japan; 6 Research Center for Genome & Medical Sciences, Tokyo Metropolitan Institute of Medical Science, Setagaya, Tokyo, Japan; Kumamoto University Faculty of Life Sciences School of Medicine: Kumamoto Daigaku Daigakuin Seimei Kagaku Kenkyubu Igakubu, JAPAN

## Abstract

We previously identified a subtype of schizophrenia (SCZ) characterized by increased plasma pentosidine, a marker of glycation and oxidative stress (PEN-SCZ). However, the genetic factors associated with PEN-SCZ have not been fully clarified. We performed a genome-wide copy number variation (CNV) analysis to identify CNVs associated with PEN-SCZ to provide an insight into the novel therapeutic targets for PEN-SCZ. Plasma pentosidine was measured by high-performance liquid chromatography in 185 patients with SCZ harboring rare CNVs detected by array comparative genomic hybridization. In three patients with PEN-SCZ showing additional autistic features, we detected a novel deletion at 7q31.1 within exons 2 and 3 of *IMMP2L*, which encodes the inner mitochondrial membrane peptidase subunit 2. The deletion was neither observed in non-PEN-SCZ nor in public database of control subjects. *IMMP2L* is one of the SCZ risk loci genes identified in a previous SCZ genome-wide association study, and its trans-populational association was recently described. Interestingly, deletions in *IMMP2L* have been previously linked with autism spectrum disorder. Disrupted IMMP2L function has been shown to cause glycation/oxidative stress in neuronal cells in an age-dependent manner. To our knowledge, this is the first genome-wide CNV study to suggest the involvement of *IMMP2L* exons 2 and 3 in the etiology of PEN-SCZ. The combination of genomic information with plasma pentosidine levels may contribute to the classification of biological SCZ subtypes that show additional autistic features. Modifying *IMMP2L* functions may be useful for treating PEN-SCZ if the underlying biological mechanism can be clarified in further studies.

## Introduction

Schizophrenia (SCZ) is a devastating psychiatric disorder with a typical onset time during adolescence or early adulthood and requires lifelong antipsychotic treatment after diagnosis. Despite intensive research efforts, treatments for SCZ remain limited to dopamine receptor D2 and 5-hydroxytryptamine receptor 2A antagonists. Novel therapeutic targets with proven efficacy have not been developed for decades.

Considering the clinical heterogeneity of SCZ [1], one of the most promising strategies for identifying novel therapeutic targets to develop personalized medicine is determining a specific treatment for each SCZ subtype defined by biological profiling, such as metabolome or transcriptome analysis [2]. We previously identified a subpopulation of SCZ characterized by high plasma pentosidine (PEN-SCZ) [3]. Pentosidine, an advanced glycation end product, is a biological marker of glycation and oxidative stress [4]. Based on these findings, we conducted a 24-week, open-label design clinical trial of pyridoxal in patients with SCZ, partially confirming its efficacy (UMIN000006398) [5]. Of the 10 participants, two patients showed marked improvements in psychotic symptoms, and one patient with a *GLO1* frameshift mutation showed considerable improvement accompanied by reduced plasma pentosidine levels. Hence, we sought to identify novel therapeutic target for PEN-SCZ by clarifying the causative factors through investigating genetic factors that confer the pathophysiology of PEN-SCZ.

The etiology of SCZ has a profound genetic component [6], with the heritability of this disease reported as 60–80% [7]. Considerable progress has been made in determining the genetic architecture of SCZ. Accumulated evidence suggests that both common and rare variants are involved in the SCZ etiology [8–12]. The Psychiatric Genomic Consortium (PGC) performed a large genome-wide association study of SCZ, in which 108 risk loci, located at genes related to synaptic networks, the immune system, and mitochondrial metabolism were found to be associated with SCZ [9]. Recently, Ikeda et al. confirmed these findings in a trans-populational manner in European, East Asian, and Japanese populations [13].

An elevated burden of rare copy number variants (CNVs) among patients with SCZ has also been well-established [14, 15]. Although common single-nucleotide polymorphisms have small individual effects (odds ratio (OR) <1.2) [9], several rare CNVs have been shown to have a much stronger impact on risk (OR <67.7) [16]. The PGC CNV Analysis Group successfully overcame the limited statistical power of small sample sizes and detected six novel loci, including Xq28, 7q11.21, and 8q22.2, in the largest genome-wide CNV study (N = 41,321) [16]. In an Asian population, Kushima et al. confirmed the increased burden of rare (≤1%) exonic CNVs using the largest sample size of the Japanese population (2,458 SCZ cases and 2,095 controls) [17].

In this study, we investigated rare (≤1%) CNVs associated with PEN-SCZ by assessing genome-wide CNV data [17] and measuring plasma pentosidine between a PEN-SCZ group and non-PEN-SCZ group.

## Materials and methods

### Participants

For both genetic and biochemical analyses, 185 unrelated, ethnically Japanese patients with SCZ and identified to harbor rare (≤1%) CNVs in the previous study [17] were assessed in this study. All patients were recruited from the Department of Neuropsychiatry, Tokyo Metropolitan Matsuzawa Hospital, Takatsuki Hospital, Takatsuki Clinic, and RIKEN Brain Science Institute located near Tokyo. Patients were diagnosed based on the DSM-IV (American Psychiatric Association) criteria for SCZ or schizoaffective disorder by the consensus of at least two experienced psychiatrists. Patients with a history of drug addiction or alcohol abuse/dependence were excluded from the study. Patients with diabetes mellitus or chronic renal disease as a comorbidity with SCZ were also excluded from the study, as these diseases may affect plasma pentosidine levels. This study was approved by the research ethics committee of the participating institutes, and written informed consent was obtained from all subjects prior to obtaining their medical records. The present study was performed in accordance with the Declaration of Helsinki. The demographics are presented in Table 1.

**Table 1:** Demographics and summary of CNVs in patients with schizophrenia with and without accumulation of plasma pentosidine.

Group	PEN-SCZ^a^	Non-PEN-SCZ^b^	p-value
Pentosidine	High	Normal	
**Number of patients**	94	91	
**Age (mean ± S.D.** ^**c**^ **)**	52.0 ± 11.2	47.0 ± 14.1	p < 0.01
**Sex (male/female)**	50/44	46/45	p = 0.68
**Age of onset (mean ± S.D.** ^**c**^ **)**	25.7 ± 9.4	25.24 ± 8.4	p = 0.37
**Average chlorpromazine equivalent doses**	1147.58 ± 864.02	716.43 ± 607.65	p < 0.001
**Plasma Pentosidine level (mean ± S.D.** ^**c**^ **) (ng/mL** ^**d**^ **)**	128.1 ± 126.0	39.8 ± 9.3	p < 0.001
**Average total CNV size (Mbp)**	3.6 ± 21.9	0.4 ± 0.5	p = 0.08
**Average total number of genes within CNVs**	92.2 ± 254.2	4.5 ± 7.6	p = 0.08

^a^Schizophrenia with accumulation of plasma pentosidine.

^b^Schizophrenia without accumulation of plasma pentosidine.

^c^Standard deviation.

^d^Cut off value; 55.2.

### CNV analysis

CNV calling was performed as described previously [17]. Briefly, NimbleGen 720K Whole-Genome Tiling arrays (probe spacing 2.5 kb; Roche NimbleGen, Madison, WI, USA) were used for genome-wide CNV screening. CNV calls were made using Nexus Copy Number software v. 7.5 (BioDiscovery, El Segundo, CA, USA) with a hidden Markov model-based approach. All genomic locations are listed in the National Center for Biotechnology Information build 36/UCSC hg18 coordinates, and Lift Genome Annotations: https://genome.ucsc.edu/cgi-bin/hgLiftOver was used to convert the genomic location from March 2006 (NCBI36/hg18) to December 2013 (GRCh38/hg38). All analyses were conducted based on gene annotation from GENCODE version 21 (http://www.gencodegenes.org/releases/21.html) [17]. Randomly selected CNVs were validated using quantitative real-time PCR combined with TaqMan copy number assays (Applied Biosystems, Foster City, CA, USA) [17]. CNVs identified in the database of healthy controls were removed using the ClinVar database (https://www.ncbi.nlm.nih.gov/clinvar/).

### Measurement of plasma pentosidine

The plasma concentration of pentosidine was measured by high-performance liquid chromatography; 55.2 ng/mL was set as the cut-off to define the PEN-SCZ and non-PEN-SCZ groups as described previously [3, 5, 18].

### Clinical characteristics of shared CNV carriers in PEN-SCZ

The clinical characteristics of PEN-SCZ cases with shared CNVs obtained through assessment of medical records included the existence of developmental delay, autistic features, epilepsy, age at onset, psychiatric symptoms, mood symptoms, antipsychotic treatment, and presence of negative symptoms.

### Statistical analysis

For demographic data, the average age, age of onset, plasma pentosidine levels, and average of chlorpromazine equivalent doses were compared by Student’s *t*-test (two-tailed). For CNV analysis, the average of total CNV sizes and total number of genes within CNVs were assessed by Student’s *t*-test. To evaluate the association between PEN-SCZ and shared exonic deletions, one-sided Fisher’s exact tests were used. If no variants were found in the two-by-two table, the OR was calculated after a 0 cell correction to reduce bias as reported previously [17].

## Results

### Exonic deletions among patients with PEN-SCZ

**Fig 1:**
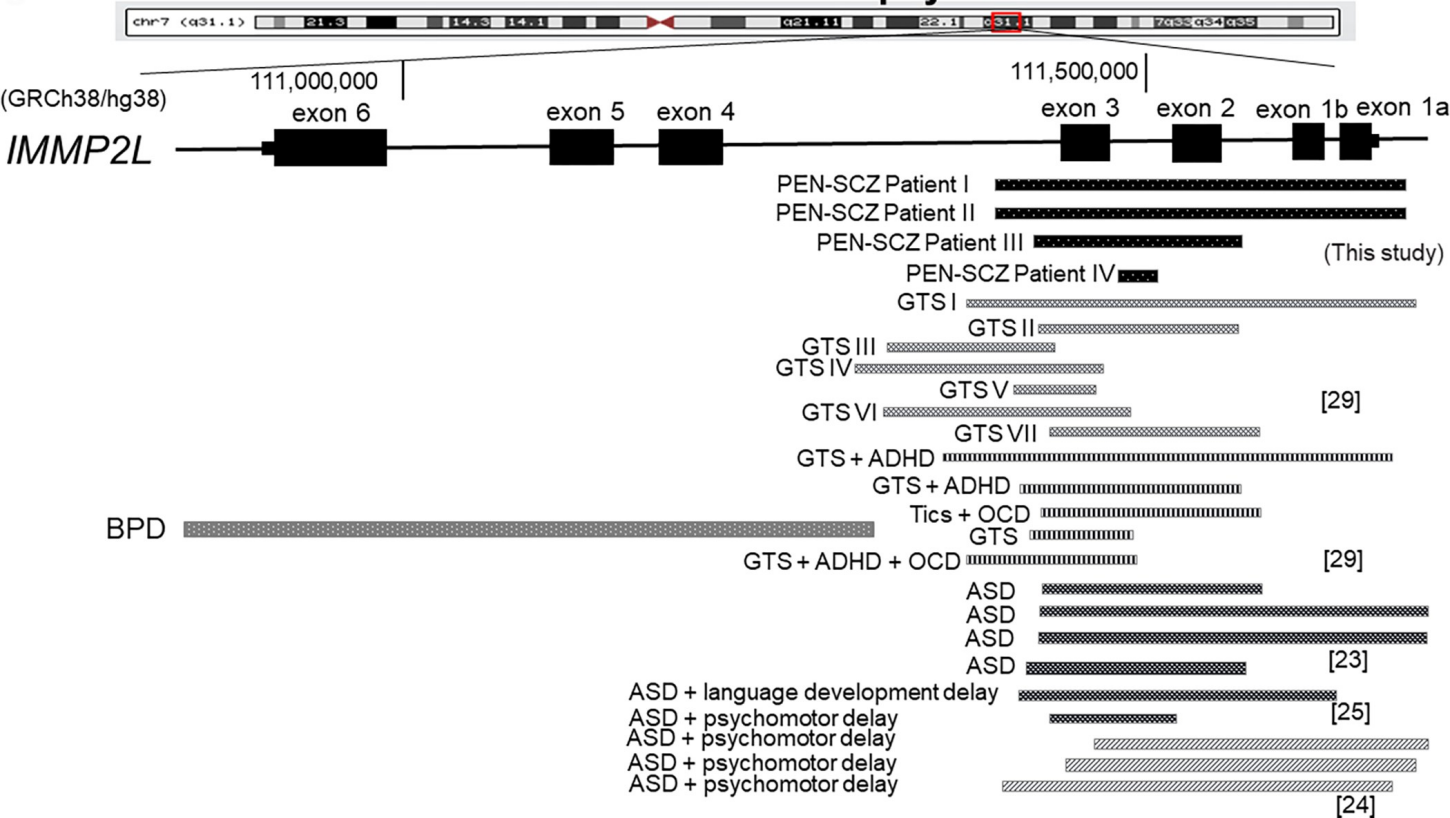
Deletions in *IMMP2L* across various neuropsychiatric disorders. A summary of the findings, including those of previous studies and this study, on *IMMP2L* deletions across neuropsychiatric disorders, is presented. Exonic deletion in *IMMP2L* tends to span from exons 1 to 3 across neuropsychiatric disorders including ASD, GTS, ADHD, and OCD. PEN-SCZ-related deletions found in this study were also restricted to exons 1–3. ASD: autism spectrum disorder; ADHD: attention-deficit hyperactivity disorder; BPD: bipolar disorder; GTS: Gilles de la Tourette syndrome; OCD: obsessive-compulsive disorder; PEN-SCZ: schizophrenia with accumulation of pentosidine in the plasma.

We investigated rare (≤1%) CNVs associated with SCZ in patients who presented with increased levels of plasma pentosidine, a biological marker for glycation and oxidative stress (PEN-SCZ), to identify novel drug targets for treating PEN-SCZ. A total of 185 patients with SCZ harboring rare CNVs was separated into two groups based on their plasma pentosidine levels (cut-off value; 55.2 ng/mL): 94 patients with PEN-SCZ and 91 patients with non-PEN-SCZ (Fig 1). The PEN-SCZ group harbored shared CNVs such as deletion in 7q31.1 and duplication in 16p13.13, which were not observed in control or non-PEN-SCZ subjects (Table 2).

**Table 2:** Rare CNVs and clinical features in patients with SCZ with and without accumulation of plasma pentosidine.

ID	Group	Demographics				CNV							Clinical manifestations
		Pentosidine (ng/mL)		Sex	Age	Location	Cytoband	Region	Size (bp)	Type	Genic	Exonic/Intronic	ASD features
								Dec. 2013 (GRCH38/hg38)					
Patient I	PEN-SCZ	83.91	high	female	22	chr7:111431428–111618385	7q31.1	chr7:111431428–111618385	186957	Del	*IMMP2L*	exons 1,2,3	Yes
Patient II	PEN-SCZ	82.96	high	male	65	chr7:111431428–111618385	7q31.1	chr7:111431428–111618385	186957	Del	*IMMP2L*	exons 1,2,3	Yes
Patient III	PEN-SCZ	64.45	high	male	38	chr7:111453103–111544709	7q31.1	chr7:111453103–111544709	91606	Del	*IMMP2L*	exons 2,3	Limited social skill
Patient IV	Non-PEN-SCZ	45.39	normal	male	60	chr7:111509431–111521305	7q31.1	chr7:111509431–111521305	11874	Del	*IMMP2L*	intron 3	No
Patient V	PEN-SCZ	89.543	high	female	56	chr16:11729544–11754368	16p13.13	chr16:11729544–11754368	24824	Dup	*TXNDC11*,	exonic	No
											*ZC3H7A*		
Patient VI	PEN-SCZ	189.34	high	female	60	chr16:11729544–11754368	16p13.13	chr16:11729544–11754368	24824	Dup	*TXNDC11*,	exonic	No
											*ZC3H7A*		
Patient VII	PEN-SCZ	106.62	high	female	46	chr16:11729544–11754368	16p13.13	chr16:11729544–11754368	24824	Dup	*TXNDC11*,	exonic	No
											*ZC3H7A*		

ASD: autism spectrum disorder; CNV: copy number variation; PEN: pentosidine; SCZ: schizophrenia; Del: deletion

**Fig 2:**
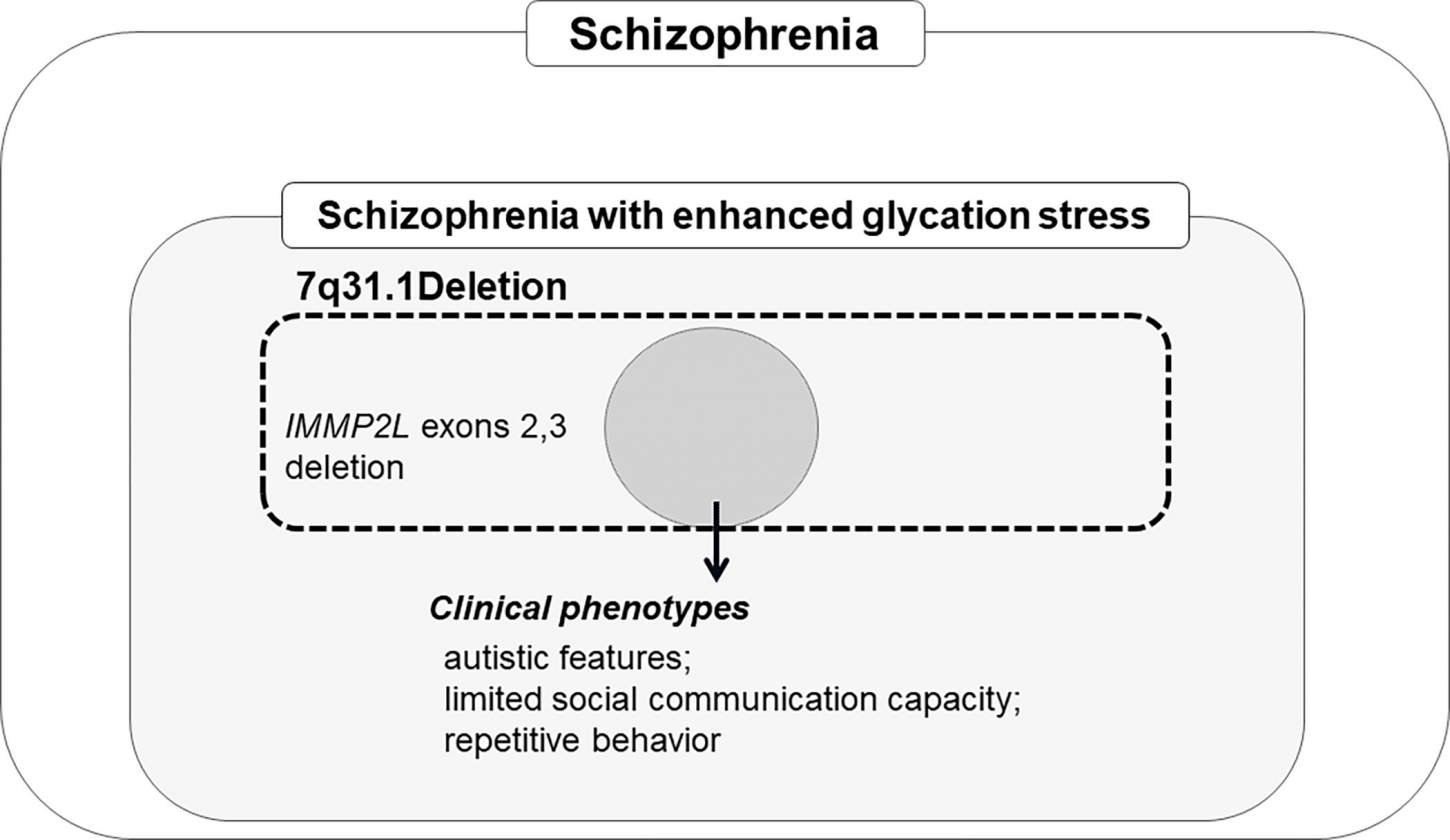
Clinical manifestation of schizophrenia with enhanced glycation stress harboring deletion in *IMMP2L* at 7q31.1. We observed a shared exonic deletion in *IMMP2L* exons 2 and 3 in three patients with SCZ presenting with accumulation of pentosidine in the plasma, although it was not significant. Patients with SCZ harboring deletions spanning exons 2 and 3 in *IMMP2L* showed autistic features, such as limited social communication skills and repetitive, obsessive behavior in addition to psychosis.

Regarding the CNV in 7q31.1, although it was not significant, deletions in *IMMP2L* exons 2 and 3 were detected in three patients with PEN-SCZ whose plasma pentosidine levels were 83.91, 82.96, and 64.45 ng/mL, respectively (p = 0.135, Table 2). Patients I and II with PEN-SCZ harbored the same deletion spanning *IMMP2L* exons 1–3, with a length of 186,957 bp from chromosome (Chr.) 7: 111,431,428 to 111,618,385. Patient III with PEN-SCZ had a deletion in *IMMP2L* exons 2 and 3 (Table 2). One non-PEN-SCZ subject had a microdeletion in 7q31.1, which included an *IMMP2L* intronic region (patient IV, Fig 2). Duplication of 16p13.13, another shared CNV in three patients with PEN-SCZ, included exons of *TXNDC11* and *ZC3H7A*, which encode the thioredoxin domain containing 11 and CCCH-type zinc finger containing 7A, respectively (Table 2).

Among the three genes disrupted by the shared CNVs, *IMMP2L* was a recurrent SCZ risk gene found in the previous SCZ genome-wide association study conducted by the PGC. Hence, we assessed whether the clinical features were shared among the three patients with PEN-SCZ harboring exonic deletions in *IMMP2L*, although its association was not significant, possibly because of the small sample size.

### Clinical characteristics of patients with PEN-SCZ harboring exonic deletions in *IMMP2L*

According to clinical records, shared clinical characteristics among the patients with PEN-SCZ were observed. For example, in addition to psychosis, the three patients harboring a deletion in *IMMP2L* exons 2 and 3 had autistic features (Table 2). Patient I was diagnosed with autism spectrum disorder (ASD) in addition to SCZ, and her social communication skills were extremely limited since childhood. Patient III also showed limited social skills since childhood and is currently considered to be a SCZ subtype for which most prominent symptoms were negative symptoms. Patient II also showed a limited social communication capability with repetitive behaviors since childhood.

## Discussion

In this study, we observed the shared 7q31.1 deletion spanning exons 2 and 3 in *IMMP2L*, known as one of the SCZ risk genes, among a subtype of SCZ with enhanced glycation and oxidative stress (PEN-SCZ), presenting with additional ASD phenotypes or limited social capability, although the association was not significant. Interestingly, deletion or duplication in other exons or introns of *IMMP2L* did not affect the plasma pentosidine level. Another CNV, a duplication at 16p13.13, was identified as a candidate for a shared genetic background in other PEN-SCZ cases, disrupting *TXNDC11* and *ZC3H7A*.

### *IMMP2L* deletions and neuropsychiatric disorders

IMMP2L, inner mitochondrial membrane peptidase 2-like, is a mitochondrial enzyme critical for glycolysis as the main pathway using glucose to respond to energy demands in the brain. *IMMP2L* knockdown in primary astrocytes leads to dysregulation of genes involved in brain development [19]. CNVs or genomic variations disrupting *IMMP2L* have been reported across neurodevelopmental psychiatric disorders, including SCZ [9, 13, 20–22], ASD [23–25], attention-deficit hyperactivity disorder (ADHD) [26, 27], and Gilles de la Tourette syndrome (GTS) [28–30]. We summarized past and present findings on *IMMP2L* deletions across neuropsychiatric disorders (Fig 1); the results suggested that behavioral phenotypes vary depending on the exons disrupted by CNVs in *IMMP2L*. Interestingly, exonic deletions in *IMMP2L* tend to span from exons 1 to 3 across various neuropsychiatric disorders, including ASD, GTS, ADHD, and OCD but excluding bipolar disorder. PEN-SCZ-related deletions found in this study also appeared to be restricted to exons 1 to 3 (Fig 1). To investigate the effect of the CNV deletion on *IMMP2L* in patients with SCZ, we examined possibility if any proteins could be produced from the allele of *IMMP2L* with the exon deletions. We found no open reading frames with enough length, as the lengths of exon 2, exon 3, and their totals are not multiple of three (that is, 137nt, 104nt, and 241nt). To assess if any compensation effect happened from the allele with no deletions, we measured *IMMP2L* mRNA in whole blood cells from patients with SCZ and a control subject. Our data indicated that the *IMMP2L* full-length form tended to be lower in patients with CNV deletion than that in a healthy individual without the CNV deletion, suggesting no compensation at the level of RNA (S1 Fig). Furthermore, in order to evaluate the possibility of loss-of-function and gain-of-function, we conducted a western blot experiment on the C-terminal antibody of IMMP2L protein. However, due to the lack of detectable *IMMP2L* expression in PBMC, definite truncated-form were not observed in a wild-type control subject.

### *IMMP2L* and schizophrenia

Notably, *IMMP2L* is an SCZ risk loci gene identified by the PGC in the previous SCZ-genome-wide association study (rank 15, p = 3.034 × 10^−13^) [9]. Ikeda et al. confirmed the trans-populational association between *IMMP2L* rs214467 and SCZ (p = 4.74 × 10^−11^) [13]. Moreover, association analysis of the 108 SCZ risk loci and cognitive functions revealed the most significant associations between rs13240464 within *IMMP2L* and “delayed recall” and “visual memory [31]. Taken together with our results from a pilot study with a small sample size, both common and rare *IMMP2L* variants may be involved in the SCZ etiology. Previous studies revealed that *IMMP2L* exon 6 transcripts were expressed across brain regions, including in the hippocampus, temporal lobe, and thalamus [29]. These transcripts are relevant to cognitive function and sensory gating and have been shown to be disrupted in patients with SCZ; however, other exons have not been fully investigated. In postmortem brain studies of SCZ, expression of glycerol-3-phosphate dehydrogenase 2, the substrate for *IMMP2L*, has been reported to be altered in the anterior cingulate cortex [32]. Although we did not observe a significant association between rare exonic deletions in *IMMP2L* and PEN-SCZ, given our small sample size, *IMMP2L* is a candidate therapeutic target in PEN-SCZ, particularly for the improvement of memory function; however, this should be evaluated in a larger sample size.

### Clinical characteristics of *IMMP2L* deletion carriers in PEN-SCZ

Patients harboring the 7q31.1 deletion, which includes *IMMP2L*, have been reported to present some repetitive behavioral phenotypes and/or obsessive-compulsive symptoms observed in ASD [25], obsessive-compulsive disorder, and GTS [28, 29]. ASD is characterized by reduced reciprocal social interaction, an impaired ability to communicate, a narrow range of interests, and repetitive behaviors. Baldan et al. identified two patients with ASD who harbored deletions in *IMMP2L* from exons 1 to 3 and in introns (Fig 2) [25]. Zhang et al. genotyped 100 trio families that included patients with ASD in a Han Chinese population and identified three cases carrying rare *IMMP2L* exonic deletions [24]. In a Danish cohort study investigating CNVs in 188 unrelated patients with Tourette syndrome, seven patients were found to have *IMMP2L* deletions, which were also restricted to exons 1–3 (Fig 1) [29].

In this study, PEN-SCZ patients I and II, who carried deletions in *IMMP2L* exons 1–3, presented with additional autistic features, including limited social communication skills in addition to psychosis (Table 2). Interestingly, a previous animal study demonstrated that *IMMP2L* knockout mice displayed behavioral changes in social interactions linked to both limited social skills in ASD and negative symptoms in SCZ [33]. Limited social skills in ASD may be broadly linked to negative symptoms in SCZ, in terms of their biological aspects associated with possibly impaired *IMMP2L* function. Supporting this notion, animal studies demonstrated dysfunction of the dopamine system in *IMMP2L* knockout mice [33]; therefore, an *IMMP2L* modulator may improve both the autistic features and limited social communication capacity.

### *IMMP2L* and glycation/oxidative stress

A mutant mouse model of *IMMP2L* has been reported to show elevated mitochondrial reactive oxygen species and an increased plasma carbonyl content, both of which reflect glycation and oxidative stress [34]. *IMMP2L* deficiency in the brain mitochondria has been shown to critically affect normal brain function [35]. In our reverse transcription-PCR experiment, gene expression levels of *IMMP2L* were lower in *IMMP2L*-deficient cases compared to wild type (S1 Fig). Immp2l deficiency has been shown to enhance production of superoxide and to have damaging effect on the mitochondrial function that may lead enhanced glycation stress [35].

Interestingly, *IMMP2L* mutant mice developed neuronal degeneration in an age-dependent manner [36]. Mitochondrial reactive oxygen species have been implicated in age-related disorders, such as neurodegenerative diseases, including Alzheimer’s disease and Parkinson’s disease [37]. Mitochondria-targeting antioxidant skQ1 has been shown to rescue age-dependent neurodegeneration in an *IMMP2L* mutant mouse model [36]. Studies are needed to determine whether the SCZ behavioral phenotype can be rescued by skQ1 in a mouse model of SCZ with enhanced glycation/oxidative stress.

In the present study, we aimed to clarify the molecular mechanisms that cause the accumulation of pentosidine. In four cases with CNV deletion, a subsequent pentosidine accumulation was expected, but it could not be confirmed in a patient IV. This observation suggests that an unknown *IMMP2L* independent molecular pathway was involved in the pentosidine alteration. Due to difficulty in re-recruiting a patient IV, we were unable to conduct further analysis. In the future, a higher number of patients must be considered for further investigation and the link between the *IMMP2L* deficiency and the mitochondrial dysfunctions or an accelerated reactive oxygen species and whether mechanisms for pentosidine linked to phenomenon occurs in patients with *IMMP2L* CNV should be clarified.

### *TXNDC11* and *ZC3H7A*

*TXNDC11* and *ZC3H7A* were also found to be affected by rare CNVs at 16p13.13. The 16p13.13 duplication has never been linked with SCZ; however, it has been reported in patients diagnosed with ASD [38], intellectual disability, developmental delay, facial asymmetry, growth deficiency, and several congenital anomalies [39]. *TXNDC11* encodes a thioredoxin domain containing 11, known as an endoplasmic reticulum-resident thioredoxin domain protein [40]. Thioredoxin is an intrinsic antioxidant system and *TXNDC11* may act as a redox regulator involved in DUOX protein folding [40]. In redox homeostasis, *TXNDX11* may be involved in the accumulation of plasma pentosidine in PEN-SCZ.

*ZC3H7A* encodes zinc finger CCCH-type containing 7A and has been shown to be a crucial regulator of immune responses [41] through the regulation of cytokine production, immune cell activation, immune homeostasis, and antiviral responses [42]. Although genetic associations between SCZ and *ZC3H7A* have never been reported, further investigation of the relationship between *ZC3H7A* and the pathophysiology of SCZ would be of interest in terms of immune system disturbance, which has also been strongly implicated in SCZ.

### Limitations

A major strength of this study is that it was an integrative study investigating CNVs with metabolomes, which provided insights into the pathophysiology of PEN-SCZ and led to the identification of a shared genetic background for this biological subtype. However, there were some limitations. First, the sample size was small; thus, further research of a larger sample size is needed to examine the involvement of *IMMP2L* in the etiology of PEN-SCZ. Second, as we only investigated rare CNVs as a genetic background, other genetic and epigenetic factors that account for enhanced glycation and oxidative stress must be investigated to clarify the whole picture of PEN-SCZ etiology. Third, patients with PEN-SCZ were significantly older and taking more medications than non-PEN-SCZ subjects. It is possible that pentosidine elevates as a function of age or medication in this population.

## Conclusions

We identified shared exonic deletion in exons 2 and 3 in *IMMP2L*, a recurrent SCZ risk locus gene, among three PEN-SCZ cases presenting with additional ASD phenotypes or limited social capability. Future research of a larger sample size is warranted to validate the association, as it was not significant possibly because of the small sample size. Experimental validation of the involvement of *IMMP2L* exonic deletion in plasma pentosidine accumulation should also be examined. Additionally, accumulation of genetic data linked with omics and phenotypic profiles would improve the definition of the biological subtypes of SCZ.

## Supporting information

S1 Fig RT-PCR analysis of *IMMP2L* levels. As shown in RT-PCR experiments, we confirmed that expression levels were lower in *IMMP2L*-deficient cases compared to wild type.
(TIF)
