# Supplementary figures and images for: Exonic deletions in IMMP2L in schizophrenia with enhanced glycation stress subtype
Source: PLoS One. 2022 Jul 1;17(7):e0270506. doi: 10.1371/journal.pone.0270506 (PMC9249242; doi:10.1371/journal.pone.0270506)

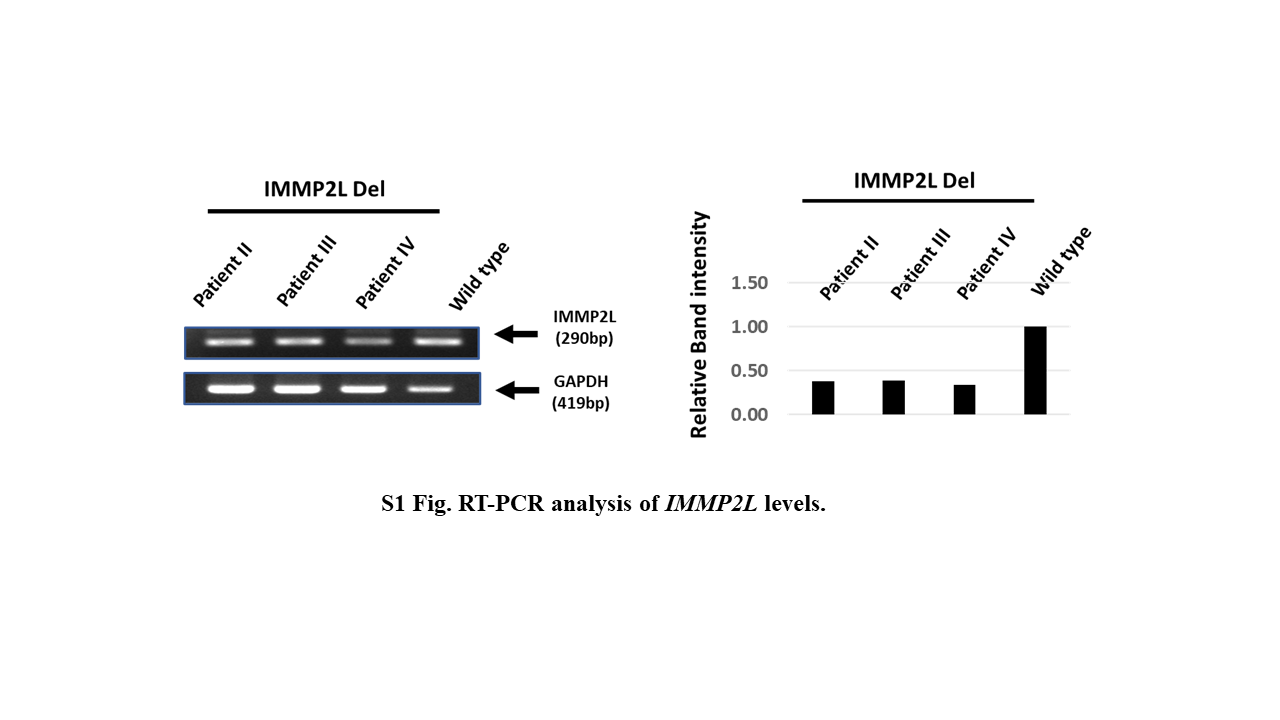

Supplement: S1 Fig — As shown in RT-PCR experiments, we confirmed that expression levels were lower in IMMP2L-deficient cases compared to wild type. (TIF) [file pone.0270506.s001.tif]
